# Serum IgG1 and IgG3 Antibodies to *Chlamydia trachomatis* Pgp3 and Hsp60 in Men of Subfertile Couples

**DOI:** 10.1093/infdis/jiaf535

**Published:** 2025-10-17

**Authors:** Tiina Holster, Päivi Joki-Korpela, Hong Yu, Robert C Brunham, Aila Tiitinen, Jorma Paavonen, Mirja Puolakkainen

**Affiliations:** Department of Obstetrics and Gynecology, University of Helsinki and Helsinki University Hospital, Helsinki, Finland; Faculty of Medicine, University of Helsinki, Helsinki, Finland; Faculty of Medicine, University of Helsinki, Helsinki, Finland; Department of Medicine, British Columbia Centre for Disease Control, University of British Columbia, Vancouver, British Columbia, Canada; Department of Medicine, British Columbia Centre for Disease Control, University of British Columbia, Vancouver, British Columbia, Canada; Faculty of Medicine, University of Helsinki, Helsinki, Finland; Faculty of Medicine, University of Helsinki, Helsinki, Finland; Faculty of Medicine, University of Helsinki, Helsinki, Finland; Virology and Immunology, University of Helsinki and Helsinki University Hospital, Helsinki, Finland

**Keywords:** *Chlamydia trachomatis*, male factor infertility, serology, Pgp3, Hsp60

## Abstract

**Background:**

Our goal was to investigate immunoglobulin G1 (IgG1) and immunoglobulin G3 (IgG3) antibody responses to *Chlamydia trachomatis* proteins Pgp3 and Hsp60 in males of subfertile couples and to explore the association of these antibodies with semen parameters and male factor infertility.

**Methods:**

Serum samples were collected from 256 male partners of subfertile couples. Serum IgG1 and IgG3 antibodies to *C trachomatis* Pgp3 and Hsp60 were measured using enzyme immunoassays. Semen samples were analyzed for volume, sperm concentration, and motility according to World Health Organization criteria.

**Results:**

Altogether, 74 (29.8%) men were seropositive to either *C trachomatis* Pgp3 IgG1 or IgG3, and 67 (27.0%) to either Hsp60 IgG1 or IgG3. *Chlamydia trachomatis* Pgp3 IgG1 and IgG3 antibodies were associated with impaired sperm motility (asthenozoospermia) (18.6% vs 6.3%, *P* = .006 for Pgp3 IgG1; and 21.4% vs 8.0%, *P* = .03 for Pgp3 IgG3). After adjusting for smoking, alcohol risk consumption, and body mass index, the association between serum *C trachomatis* Pgp3 IgG1 seropositivity and asthenozoospermia remained statistically significant (odds ratio, 3.0 [95% confidence interval, 1.12–8.01]; *P* = .03). The presence of Hsp60 IgG1 antibody was associated with a higher teratozoospermia index (1.47 ± 0.15 vs 1.39 ± 0.16; *P* = .001).

**Conclusions:**

Our results suggest that prior *Chlamydia trachomatis* infection, as indicated by Pgp3 seropositivity, may negatively impact male fertility potential by affecting sperm motility and morphology.

## Teaser

The study examined IgG1 and IgG3 antibody responses to *Chlamydia trachomatis* proteins Pgp3 and Hsp60 in subfertile men. The findings suggest that prior chlamydial infection may impair male fertility by adversely affecting sperm motility and morphology.

*Chlamydia trachomatis* is one of the most common bacteria causing sexually transmitted infections, with 128.5 million new cases occurring annually among young adults [1]. Up to 50% of male chlamydial infections are asymptomatic, and in those with symptoms, the most common manifestation is urethritis [2]. In young men, *C trachomatis* is one of the most common etiological agents of epididymitis and which may affect fertility due to chronic inflammation and obstruction, especially when both testes are affected [3, 4]. Chlamydia may also cause epithelial damage by impairing spermatogenesis at the various levels of spermatozoa development [5] or inducing immune responses that destroy sperm cells through apoptosis [6]. Male infertility has remained a neglected field in reproductive health, although male factor etiology may contribute for 40%–50% of fertility problems [7] and up to 15% of idiopathic cases of male subfertility are estimated to be related to infectious causes [8]. Also, lifestyle factors, including cigarette smoking [9] and obesity [10], have been recognized as detrimental to male reproductive health. Alcohol consumption may also have a negative impact on sperm parameters, but the results have been controversial [11].

Most individuals with *C trachomatis* infection develop serum immunoglobulin G (IgG) antibodies, which can persist for years and serve as a marker of past chlamydial infection. In particular, the antibody response to the highly immunogenic, plasmid-encoded protein Pgp3 has demonstrated high specificity and sensitivity for detecting ongoing or past infection in both women and men [12–14]. The presence of *C trachomatis* Pgp3-specific IgG1 and IgG3 antibodies has been strongly associated with tubal factor infertility (TFI) in subfertile women [15]. However, the significance of this immune response in male infertility has not been studied before.

IgG1 and IgG3 are the predominant human serum antibody subclasses produced in response to *C trachomatis* infection [16]. IgG1 is involved in both early and late phases of the immune response, whereas IgG3 is more prominent during the early phase but declines more rapidly [17]. In our current study, we examined the presence of serum *C trachomatis* Pgp3 and Hsp60 IgG1 and IgG3 antibodies among subfertile men and explored the association of antibodies with semen parameters and male factor infertility. Measuring these subclasses separately is particularly informative, as IgG1 may indicate past infection due to its longer lifespan, while IgG3 antibody is more reflective of recent exposure [17].

## METHODS

### Study Population

**Figure 1.**
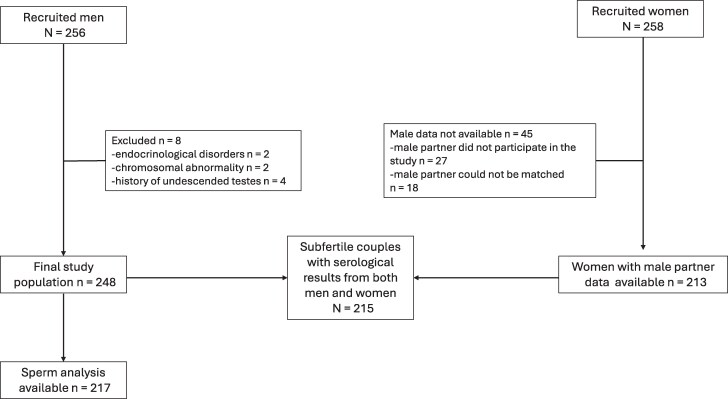
Flowchart of the study population.

Serum samples for serological analysis were collected from 256 male partners of subfertile couples referred for infertility investigations at Helsinki University Hospital, Reproductive Medicine Unit, during July 2007—December 2010. Their female partners had been recruited for our prior studies examining immune responses to *C trachomatis* in subfertile women [18–20]. For the present study, we identified 215 females and matched their serological results with their male partners’ data (Figure 1). Clinical information and fertility evaluation results were collected from the patient registers.

Men with known causes of impaired sperm quality, including endocrinological disorders (n = 2), chromosomal anomalies (n = 2), or history of undescended testes (n = 4), were excluded from the analysis. Ultimately, the study population comprised 248 men from couples with at least 1 year of subfertility.

Semen analysis data were available for 217 men. Five couples conceived spontaneously, and 1 couple divorced before completing infertility investigations. Data were unavailable for 25 men whose semen samples had been analyzed at a private clinic before their admission to the hospital's outpatient clinic (Figure 1).

Semen samples were collected as part of a routine fertility evaluation and analyzed for volume, sperm concentration (count/mL), and motility (classified as A + B + C + D), according to World Health Organization (WHO) criteria [21]. In this classification, categories A and B represent progressive motility, C indicates nonprogressive motility, and D denotes immotile sperm.

Oligozoospermia was defined as a sperm concentration <15 million/mL, while asthenozoospermia was characterized by a progressive motile spermatozoa (A + B) percentage of <32% [21]. Sperm morphology was assessed in 191 samples with teratozoospermia, defined as ≤4% of spermatozoa exhibiting normal morphology. The teratozoospermia index (TZI), which reflects the average number of abnormalities per abnormal spermatozoon [22], was available for 165 samples. To evaluate the presence of antisperm antibodies (ASAs), the mixed antiglobulin reaction test was performed on 181 samples.

### Serology

Serum samples were collected at the first outpatient clinic visit and stored at −20°C until analyzed. IgG1 and IgG3 antibody responses to *C trachomatis* Pgp3 and Hsp60 were analyzed by enzyme immunoassay as described previously [14, 15]. In brief, the purified recombinant Pgp3 and Hsp60 proteins were obtained from Biomatik (Cambridge, Ontario, Canada) [14]. The proteins were coated to microtiter plates (Costa Assay 96-well plate, Corning). After washing and blocking, sera diluted 1:32 were applied to wells in triplicate. After washing, 1:500 diluted alkaline phosphatase (AP)–conjugated anti-human IgG secondary antibodies were added. To test for IgG1 antibodies, Mouse Anti-Human IgG1, Fc Fragment Specific Alkaline Phosphatase Conjugate (HP6069) (Millipore Sigma) and Mouse Anti-Human IgG1 Hinge-AP (4E3) (Southern Biotech) were pooled. To test for IgG3 antibodies, Mouse Anti-Human IgG3 Hinge-AP (HP6050) (Southern Biotech) was used. Finally, the plates were again washed, and *p*-NPP (Sigma: 1 tablet of *p*-nitrophenyl phosphate plus 1 tablet of Tris in 20 mL H_2_O) was added and incubated at room temperature in the dark. The optical density at 405 nm was read at exactly 30 minutes after addition of substrate. The cut-off values were based on the absorbance values (mean ± 2 standard deviations [SD]) obtained using serum specimens of individuals with no *C trachomatis* antibody detectable by microimmunofluorescence test [15]. The cut-off was 0.263 for Pgp3 IgG1, 0.140 for Pgp3 IgG3, 0.262 for Hsp60 IgG1, and 0.145 for Hsp60 IgG3.

### Statistical Analysis

The χ^2^ test was used for the analysis of categorical data, and continuous variables were compared by Mann–Whitney *U*-test and Kruskal–Wallis test. *P* < .05 was considered statistically significant. Logistic regression was used to estimate the probability of the categorical variables and to adjust the outcomes for smoking, alcohol risk consumption, and body mass index (BMI). Statistical analysis was performed using IBM SPSS statistical software version 29.0 (SPSS Inc, Chicago, IL, USA).

## RESULTS

**Figure 2.**
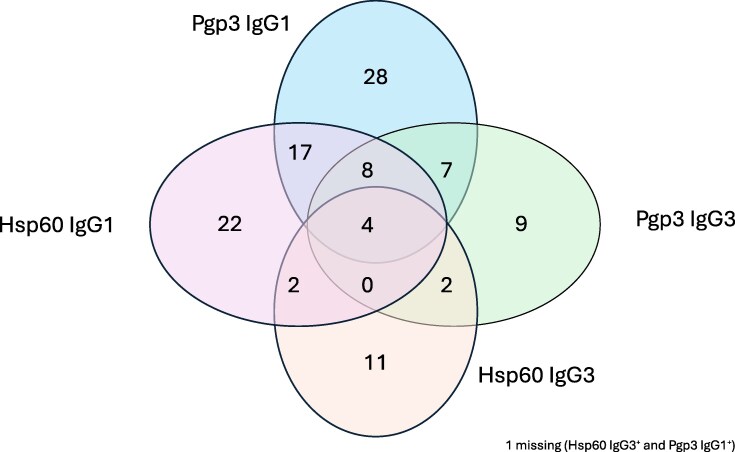
Overlap between *Chlamydia trachomatis* Pgp3 immunoglobulin G1 (IgG1), Pgp3 immunoglobulin G3 (IgG3), Hsp60 IgG1, and Hsp60 IgG3 seropositivity.

Of the 248 males of infertile couples, 65 (26.2%) had *C trachomatis* Pgp3 IgG1 antibodies and 30 (12.1%) had Pgp3 IgG3 antibodies. *Chlamydia trachomatis* Hsp60 IgG1 antibody test was positive in 53 (21.4%) men, and Hsp60 IgG3 in 20 (8.1%) men. Altogether 74 (29.8%) men were seropositive to either Pgp3 IgG1 or IgG3, and 67 (27.0%) to either Hsp60 IgG1 or IgG3 (Figure 2).

The mean age of the men was 32.6 years (SD, 5.1 years) with no statistically significant difference between seropositive and seronegative individuals. *Chlamydia trachomatis* Pgp3 IgG1 and IgG3 antibodies were associated with a history of self-reported chlamydial infection (19.0% vs 6.8%, *P* = .006 for Pgp3 IgG1; and 23.3% vs 8.1%, *P* = .01 for Pgp3 IgG3). Men with serum *C trachomatis* Pgp3 IgG1 antibodies had a higher BMI than the seronegative individuals (mean [SD], 26.9 [4.9] kg/m^2^ vs 25.5 [3.1] kg/m^2^; *P* = .04). Additionally, the presence of Pgp3 IgG1 antibodies was associated with smoking (44.4% vs 27.3%; *P* = .01), while Hsp60 IgG1 seropositivity was linked to alcohol risk consumption (≥14 doses/week) (19.6% vs 7.3%; *P* = .01). The basic characteristics of the study population are presented in Table 1.

**Table 1.** Baseline Characteristics of the Study Population

Variable	Pgp3 IgG1 Positive (n = 65)	Pgp3 IgG1 Negative (n = 183)	*P* Value	Pgp3 IgG3 Positive (n = 30)	Pgp3 IgG3 Negative (n = 218)	*P* Value	Hsp60 IgG1 Positive (n = 53)	Hsp60 IgG1 Negative (n = 195)	*P* Value	Hsp60 IgG3 Positive (n = 20)	Hsp60 IgG3 Negative (n = 228)	*P* Value
Age, y, mean (SD [range])	32.6 (5.8 [18–49])	32.6 (4.8 [22–48])	.96	33.8 (6.9 [18–49])	32.4 (4.7 [22–48])	.33	33.7 (6.2 [22–49])	32.3 (4.6 [18–48])	.11	32.1 (5.6 [22–47])	32.6 (5.0 [18–49])	.48
BMI, kg/m^2^, mean (SD [range])^a^	26.9 (4.9 [19–50])	25.5 (3.1 [18–35])	.**04**	27.6 (5.8 [20–50])	25.7 (3.3 [18–35])	.12	27.0 (4.5 [21–50])	25.6 (3.4 [18–35])	.05	26.7 (6.5 [21–50])	25.8 (3.4 [18–35])	.76
Prior chlamydia^a^			.**006**			.**01**			.17			.47
Yes (n = 24)	12 (19.0)	12 (6.8)		7 (23.3)	17 (8.1)		8 (15.1)	16 (8.6)		1 (5.3)	23 (10.5)	
No (n = 215)	51 (81.0)	164 (93.2)		23 (76.7)	192 (91.9)		45 (84.9)	170 (91.4)		18 (94.7)	197 (89.5)	
Prior other STI (herpes, gonorrhea, HPV)^a^			.79			.26			1.00			.64
Yes (n = 18)	4 (6.3)	14 (8.0)		4 (13.3)	14 (6.7)		4 (7.5)	14 (7.5)		2 (10.5)	16 (7.3)	
No (n = 221)	59 (93.7)	162 (92.0)		26 (86.7)	195 (93.3)		49 (92.5)	172 (92.5)		17 (89.5)	204 (92.7)	
Smoking^a^			.**01**			.30			.09			.32
Yes (n = 76)	28 (44.4)	48 (27.3)		12 (40.0)	64 (30.6)		22 (41.5)	54 (29.0)		8 (42.1)	68 (30.9)	
No (n = 163)	35 (55.6)	128 (72.7)		18 (60.0)	145 (69.4)		31 (58.5)	132 (71.0)		11 (57.9)	152 (69.1)	
Alcohol risk consumption^b^			.33			.17			.**01**			.70
Yes (n = 17)	8 (13.3)	15 (8.9)		5 (17.2)	18 (9.0)		10 (19.6)	13 (7.3)		1 (5.3)	22 (10.5)	
No (n = 203)	52 (86.7)	153 (91.1)		24 (82.8)	181 (91.0)		41 (80.4)	164 (92.7)		18 (94.7)	187 (89.5)	

Data are presented as No. (column %) unless otherwise indicated. Statistically significant *P*-values are in bold.

Abbreviations: BMI, body mass index; HPV, human papillomavirus; IgG1, immunoglobulin G1; IgG3, immunoglobulin G3; SD, standard deviation; STI, sexually transmitted infection.

^a^Data missing in 9 patients.

^b^Data missing in 27 patients.

The semen parameters of the study population are shown in Table 2. *Chlamydia trachomatis* Pgp3 IgG1 and IgG3 antibodies were associated with asthenozoospermia (18.6% vs 6.3%, *P* = .006 for Pgp3 IgG1; and 21.4% vs 8.0%, *P* = .03 for Pgp3 IgG3). Furthermore, the presence of Hsp60 IgG1 antibody was associated with a higher TZI (1.47 ± 0.15 vs 1.39 ± 0.16; *P* = .001). After adjusting for potential confounders, including smoking, alcohol risk consumption, and BMI, the association between serum *C trachomatis* PgP3 IgG1 seropositivity and asthenozoospermia remained statistically significant (odds ratio [OR], 3.0 [95% confidence interval {CI}, 1.12–8.01]; *P* = .03).

**Table 2.** Key Semen Parameters of the Study Population. Semen Analysis Data were Available for 217 Men

Variable	Pgp3 IgG1 Positive (n =59)	Pgp3 IgG1 Negative (n = 158)	*P* Value	Pgp3 IgG3 Positive (n = 28)	Pgp3 IgG3 Negative (n = 189)	*P* Value	Hsp60 IgG1 Positive (n = 48)	Hsp60 IgG1 Negative (n = 169)	*P* Value	Hsp60 IgG3 Positive (n = 18)	Hsp60 IgG3 Negative (n = 199)	*P* Value
Sample volume (mL)												
Mean (SD [range])	3.6 (1.4 [1.0–7.5])	3.1 (1.5 [0.5–8.5])	.**01**	3.1 (1.3 [1.5–6.5])	3.2 (1.5 [0.5–8.5])	.77	3.5 (1.7 [1.5–8.5])	3.1 (1.5 [0.5–8.5])	.27	3.5 (1.7 [2.0–7.0])	3.2 (1.5 [0.5–8.5])	.68
Sperm concentration (million/mL)												
Mean (SD [range])	48.5 (34.1 [0.6–151.0])	53.4 (33.7 [0.2–180.0])	.21	49.7 (37.6 [5.0–151.0])	52.4 (33.3 [0.2–180.0])	.51	49.0 (37.4 [2.8–176.0])	52.9 (33.0 [0.2–180.0])	.28	48.4 (34.3 [6.0–151.0])	52.3 (33.8 [0.2–180.0])	.51
Oligozoospermia (≤ 15 million/mL)			.95			.26			1.00			.18
Yes (n = 18)	5 (8.5)	13 (8.2)		4 (14.3)	14 (7.4)		4 (8.3)	14 (8.3)		3 (16.7)	15 (7.5)	
No (n = 199)	54 (91.5)	145 (91.8)		24 (85.7)	175 (92.6)		44 (91.7)	155 (91.7)		15 (83.3)	184 (92.5)	
Progressive motility (A + B, %)												
Mean (SD [range])	53.9 (16.6 [20.0–89.0])	57.8 (14.3 [8.0–89.0])	.13	54.0 (19.9 [8.0–89.0])	57.1 (14.1 [9.0–87.0])	.38	55.2 (15.9 [20–80])	57.1 (14.8 [8–89])	.73	54.4 (17.6 [8–73])	56.9 (14.8 [9–89])	.90
Total motility (A + B + C, %)												
Mean (SD [range])	64.4 (19.2 [33–152])	64.8 (12.7 [11–91])	.19	61.4 (17.4 [30–91])	65.0 (14.4 [11–152])	.25	63.6 (19.1 [33–152])	64.6 (13.4 [11–100])	.33	63.9 (14.6 [30–73])	64.6 (14.9 [11–152])	.66
Asthenozoospermia (A + B ≤32%)			.**006**			.**02**			.19			.39
Yes (n = 21)	11 (18.6)	10 (6.3)		6 (21.4)	15 (7.9)		7 (14.6)	14 (8.3)		3 (14.3)	18 (9.0)	
No (n = 196)	48 (81.4)	148 (93.7)		22 (78.6)	174 (92.1)		41 (85.4)	155 (91.7)		15 (83.3)	181 (91.0)	
Teratozoospermia (normal morphology ≤4%)^a^			.17			.75			.07			.47
Yes (n = 112)	34 (66.7)	78 (55.7)		16 (61.5)	96 (58.2)		29 (70.7)	83 (55.3)		12 (66.7)	100 (57.8)	
No (n = 79)	17 (33.3)	62 (44.3)		10 (38.5)	69 (41.8)		12 (29.3)	67 (44.7)		6 (33.3)	73 (42.2)	
TZI^b^												
Mean (SD [range])	1.43 (0.17 [1.18–1.89])	1.40 (0.15 [1.20–2.29])	.28	1.42 (0.13 [1.22–1.76])	1.41 (0.16 [1.18–2.29])	.29	1.47 (0.15 [1.28–1.85])	1.39 (0.16 [1.18–2.29])	.**001**	1.40 (0.14 [1.24–1.77])	1.41 (0.16 [1.18–2.29])	.86
MAR test^c^			1.00			.20			.31			1.00
Positive (n = 13)	3 (6.7)	10 (7.4)		3 (13.6)	10 (6.3)		1 (2.6)	12 (8.4)		1 (6.7)	12 (7.2)	
Negative (n = 168)	42 (93.3)	126 (92.6)		19 (86.4)	149 (93.7)		37 (97.4)	131 (91.6)		14 (93.3)	154 (92.8)	

Data are presented as No. (column %) unless otherwise indicated. Statistically significant *P*-values are in bold.

Abbreviations: IgG1, immunoglobulin G1; IgG3, immunoglobulin G3; MAR, mixed antiglobulin reaction; SD, standard deviation; TZI, teratozoospermia index.

^a^Data missing from 26 patients.

^b^Data missing from 52 patients.

^c^Data missing from 36 patients.

Table 3 presents the clinical characteristics of the couples. The presence of *C trachomatis* Pgp3 IgG1 antibody in female serum was associated with Hsp60 IgG1 seropositivity in their male partners (56.5% vs 29.3%; *P* < .001). Additionally, female Pgp3 IgG3 seropositivity was linked to male Hsp60 IgG1 (28.3% vs 12.6%; *P* = .01) and Hsp60 IgG3 antibodies (38.9% vs 13.8%; *P* = .006). Male factor infertility (*International Classification of Diseases, Tenth Revision* [ICD-10] code N97.4) as the main infertility diagnosis was more common in the couples where the male had serum *C trachomatis* Pgp3 IgG1 (23.7% vs 12.3%; *P* = .04) or IgG3 (28.6% vs 13.4%; *P* = .04) antibodies. The likelihood of having a male factor infertility diagnosis was >2-fold if the male partner had either IgG1 or IgG3 chlamydial Pgp3 antibodies (OR, 2.4 [95% CI, 1.1–5.0]; *P* = .02]. TFI diagnosis (ICD-10 code N97.1), defined as occlusion in at least 1 fallopian tube, was associated with male *C trachomatis* Hsp60 IgG3 seropositivity (26.3% vs 5%, *P* < .001). Table 4 represents the various combinations of Pgp3 antibodies among the partners of subfertile couples and their associations to clinical variables. The duration of infertility was significantly shorter when both male and female were Pgp3 IgG1 and IgG3 negative (mean, 1.70 [SD, 1.15; range, 0.5–10.0]).

**Table 3.** Presence of Serum *Chlamydia trachomatis* Antibodies and Selected Background Variables of the Subfertile Couples. Serological Results from Both Men and Women were Available for 215 Couples

Variable	Male Pgp3 IgG1 Positive (n = 65)	Male Pgp3 IgG1 Negative (n = 183)	*P* Value	Male Pgp3 IgG3 Positive (n = 30)	Male Pgp3 IgG3 Negative (n = 218)	*P* Value	Male Hsp60 IgG1 Positive (n = 53)	Male Hsp60 IgG1 Negative (n = 195)	*P* Value	Male Hsp60 IgG3 Positive (n = 20)	Male Hsp60 IgG3 Negative (n = 228)	*P* Value
Seropositivity of female partner												
Pgp3 IgG1 (n = 75)	25 (33.3)	50 (66.7)	.07	11 (14.7)	64 (85.3)	.52	26 (34.7)	49 (65.3)	**< .001**	10 (13.3)	65 (86.7)	.06
Pgp3 IgG3 (n = 34)	13 (38.2)	21 (61.8)	.07	7 (20.6)	27 (79.4)	.13	13 (38.2)	21 (61.8)	.**01**	7 (20.6)	27 (79.4)	.**006**
Hsp60 IgG1 (n = 40)	9 (22.5)	31 (77.5)	.59	6 (15.0)	34 (85.0)	.62	7 (17.5)	33 (82.5)	.49	5 (12.5)	35 (87.5)	.31
Hsp60 IgG3 (n = 17)	6 (35.3)	11 (64.7)	.35	4 (23.5)	13 (76.5)	.16	3 (17.6)	14 (82.4)	1.00	3 (17.6)	14 (82.4)	.16
Type of infertility			.70			.88			.81			.11
Primary (n = 154)	39 (25.3)	115 (74.7)		19 (12.3)	135 (87.8)		33 (21.4)	121 (78.6)		10 (6.5)	144 (93.5)	
Secondary (n = 61)	17 (27.9)	44 (72.1)		8 (13.1)	53 (86.9)		14 (23.0)	47 (77.0)		8 (13.1)	53 (86.9)	
Infertility diagnosis of the couple^a^												
Endometriosis (n = 28)	5 (17.9)	23 (82.1)	.26	3 (10.7)	25 (89.3)	1.00	2 (7.1)	26 (92.9)	.05	3 (10.7)	25 (89.3)	.72
Anovulation (n = 41)	10 (24.3)	31 (75.6)	.72	4 (9.8)	37 (90.2)	.79	5 (12.2)	36 (87.8)	.09	1 (2.4)	40 (97.6)	.13
Tubal factor (n = 15)	3 (20.0)	12 (80.0)	.76	4 (26.7)	11 (73.3)	.1	4 (26.7)	11 (73.3)	.75	5 (33.3)	10 (66.7)	**< .001**
Male factor (n = 34)	14 (41.2)	20 (58.8)	.**04**	8 (23.5)	26 (76.5)	.**04**	9 (26.5)	25 (73.5)	.50	4 (11.8)	30 (88.2)	.47
Unexplained (n = 96)	27 (28.2)	69 (71.8)	.66	9 (9.4)	87 (90.6)	.20	28 (29.2)	68 (70.8)	.**02**	7 (7.3)	89 (92.7)	.46
Duration of infertility, y												
Mean (SD [range])	2.14 (1.65 [0.5–9.0])	1.78 (1.17 [0.5–10.0])	.08	2.33 (2.14 [0.5–9.0])	1.80 (1.14 [0.5–10.0])	.31	1.97 (1.73 [0.5–9.0])	1.84 (1.18 [0.5–10.0])	.88	2.46 (2.02 [1.0–9.0])	1.82 (1.22 [0.5–10.9])	.20

Data are presented as No. (row %) unless otherwise indicated. Statistically significant *P*-values are in bold.

Abbreviations: IgG1, immunoglobulin G1; IgG3, immunoglobulin G3; SD, standard deviation.

^a^Data missing in 4 couples.

**Table 4.** Various Combinations of *Chlamydia trachomatis* Pgp3 Antibodies Among the Male and Female Partners of Subfertile Couples and Their Associations With Selected Clinical Variables

Antibody	Pgp3 IgG1			Pgp3 IgG3			Pgp3 IgG1 and/or IgG3		
Couple's Serostatus (N = 215)	Male^+^/Female^−^ (n = 32)	Male^−^/Female^+^ (n = 48)	Male^−^/Female^−^ (n = 101)	Male^+^/Female^−^ (n = 20)	Male^−^/Female^+^ (n = 27)	Male^−^/Female^−^ (n = 157)	Male^+^/Female^−^ (n = 44)	Male^−^/Female^+^ (n = 60)	Male^−^/Female^−^ (n = 90)
History of infertility, y									
Mean (SD [range])	1.96 (0.94 [0.7−5.0])	1.80 (0.82 [1.0−4.5])	**1.77 (1.30 [0.5**−10.0])*	1.98 (1.80 0.5−9.0])	1.76 (0.85 [1.0−4.0])	1.81 (1.20 [0.5−10.0])	2.00 (1.40 [0.5−9.0])	1.75 (0.83 [1.0−4.5])	**1.70 (1.15 [0.5**−10.0])*
Primary	25 (78.1)	34 (70.8)	72 (71.3)	18 (90.0)	13 (48.1)***	117 (74.5)	36 (81.8)	40 (66.7)	64 (71.1)
Secondary	7 (21.9)	14 (29.2)	29 (28.7)	2 (10.0)	**14 (51.9)**	40 (25.5)	8 (18.2)	20 (33.3)	26 (28.9)
TFI, OR (95% CI)	0.56 (.07−4.7)	**3.09 (1.1**−9.0)*	0.48 (.16−1.5)	1.61 (.34−7.74)	**4.14 (1.29**−13.28)**	**0.20 (.07**−.59)***	0.59 (.13−2.66)	2.41 (.83−6.97)	0.32 (.09−1.16)
Male factor infertility, OR (95% CI)	2.14 (.82−5.57)	0.49 (.16−1.5)	0.97 (.43−2.19)	1.81 (.56−5.91)	0.55 (.11−2.56)	0.56 (.24−1.30)	**2.54 (1.07**−6.01)*	0.53 (.19−1.46)	0.56 (.24−1.30)
Unexplained infertility, OR (95% CI)	1.33 (.62−2.85)	0.55 (.28−1.10)	1.30 (.74−2.27)	0.67 (.25−1.78)	0.83 (.35−1.97)	1.65 (.86−3.15)	0.98 (.50−1.94)	0.61 (.33−1.14)	1.20 (.69−2.10)

Statistically significant *P*-values are in bold.

Abbreviations: CI, confidence interval; IgG1, immunoglobulin G1; IgG3, immunoglobulin G3; OR, odds ratio; SD, standard deviation; TFI, tubal factor infertility.

*P < .05; **P < .01; ***P < .005.

## DISCUSSION

This study investigated the prevalence and clinical relevance of serum *C trachomatis* Pgp3 and Hsp60 IgG1 and IgG3 antibodies among male partners of subfertile couples. We found that prior chlamydial infection, as indicated by Pgp3 antibody seropositivity, was associated with male factor infertility, particularly with impaired sperm motility.

While the association between *C trachomatis* infection and female infertility is well established, its long-term impact on male fertility remains less clear. To our knowledge, the present study is the first to specifically examine serum *C trachomatis* Pgp3 IgG1 and IgG3 antibodies among subfertile men and their relationship with semen quality. Both Pgp3 IgG1 and IgG3 antibodies were strongly associated with self-reported prior infection, in line with earlier findings [13, 15], underscoring the immunogenic relevance of Pgp3 as a serological marker of chlamydial history. These antibodies were also associated with male factor infertility and impaired progressive motility of sperm (asthenozoospermia). Additionally, higher BMI and smoking were associated with Pgp3 antibody positivity, and heavy alcohol consumption was associated with Hsp60 IgG1 positivity. These parameters may reflect general risk-taking behavior. Of interest, also alcohol consumption [11], smoking [9], and higher BMI [10] have a dose-dependent effect on sperm quality, yet Pgp3 IgG positivity was an independent risk factor for male infertility due to impaired sperm motility.

Previous serological studies support an association between *C trachomatis* infection and male infertility. Serum *C trachomatis* IgG antibodies to a major outer membrane protein (MOMP)–derived peptide were more frequently detected in infertile men than in fertile controls, and their presence was linked to lower sperm count [23]. Additionally, antibodies to *C trachomatis* MOMP in men have been linked to reduced couple pregnancy rates, independent of the female partner's serostatus [24, 25]. In our study, Hsp60 IgG1 antibody was associated with higher TZI, indicating higher average number of abnormalities per abnormal spermatozoon compared to seronegative males, suggesting that chronic chlamydial infection might be associated with abnormal semen morphology. No association between Hsp60 antibodies and sperm motility was noted. Conversely, Idahl et al found that male *C trachomatis* Hsp60 IgG antibodies were associated with reduced percentage of motile spermatozoa [26]. In our study, Hsp60 IgG antibodies were associated with heavy alcohol consumption, known to influence sperm quality as such [11]. Higher BMI and smoking, known to affect quality of sperm, were more common among Hsp60 IgG1 positives, but this did not reach statistical significance. The likely explanation includes that Hsp60 is highly conserved across bacterial and even human species, and cross-reactivity has been demonstrated earlier [27, 28]. Moreover, Karinen et al showed that antibody to Hsp60 was less common in the male partners of subfertile couples than in their fertile controls, and among male partners of subfertile couple, especially among smokers, serum antibody levels to Hsp60 antigens were lower than in the controls [29]. The reason for this discrepancy remains unknown.

Most previous studies have focused on the role of acute chlamydial infection in male infertility. In our cohort, nucleic acid amplification tests (NAATs) for *C trachomatis* were not performed on male participants. However, all of the female partners had been tested as part of their infertility screening and were found negative [18–20]. Acute infection in men was therefore unlikely. Studies evaluating the influence of acute *C trachomatis* infection, as indicated by antigen, culture, or NAAT positivity, on sperm parameters have yielded inconsistent results regarding ejaculate volume, sperm concentration, sperm motility, and morphology [30–32].

Evidence on how *C trachomatis* contributes to male infertility remains limited. Genitourinary infections and chronic inflammation, including those caused by *C trachomatis*, may impair sperm motility through the induction of reactive oxygen species (ROS) [3]. Lifestyle factors such as smoking, poor diet, obesity, and alcohol use also reduce motility [33, 34]. In our study, smoking was associated with *C trachomatis* Pgp3 IgG1 seropositivity, and seropositive men had higher BMI. Notably, both Pgp3 IgG1 and IgG3 antibodies were independently associated with asthenozoospermia, even after adjusting for smoking and BMI.

Upper genital tract infections like prostatitis and epididymitis—commonly attributed to *C trachomatis*—are prevalent in young men and may affect fertility [35]. In vitro studies show that *C trachomatis* can directly reduce sperm motility and viability [36–38], while in vivo data suggest that it damages Sertoli cells and disrupts spermatogenesis [39]. The bacterium's intracellular persistence may promote chronic inflammation, ROS production, and apoptosis [8, 31, 40]. Inflammation can lead to scarring and obstruction in the male reproductive tract [4], though obstructive male factor infertility is rare and typically leads to azoospermia. More likely, immune responses damage the epithelium and impair spermatogenesis [2]. Although *C trachomatis* infection has also been linked to production of ASAs [2, 41], our study found no correlation between serum Pgp3 or Hsp60 antibodies and ASAs.

Our study showed that *C trachomatis* infection in the male partner can reduce fertility potential of a couple. When we analyzed the presence of serum *C trachomatis* Pgp3 antibodies among both partners of the subfertile couples, Pgp3 IgG1 and IgG3 positivity of the female partner was strongly linked to TFI. This association between chlamydial infection and TFI has been well documented in many studies [42], and in our earlier study we also showed that Pgp3 IgG1, in particular, is a sensitive marker of TFI [15]. In the current study, couples in which both partners were seronegative for Pgp3 IgG3 were less often diagnosed with TFI. Similarly, couples who were both seronegative for Pgp3 IgG1 had a shorter history of infertility. However, the etiology of impaired fertility is often multifactorial as couples may present with multiple contributing causes of subfertility. Our findings confirm that *C trachomatis* is potentially capable to cause reproductive harm in both sexes, affecting not only the female reproductive system, but also male fertility by impairing sperm quality.

Limitations of our study include its retrospective nature and inability to evaluate antibody persistence over time. As a result, we could not determine whether *C trachomatis* infection leading to antibody positivity was transmitted within relationships, or if partner selection was more common among individuals engaging in high-risk behavior. Additionally, we were unable to assess changes in semen quality over time. Although environmental factors may influence male infertility, they were not included in this analysis. Despite these limitations, the study’s strengths include consistent eligibility criteria, outcome measures, and IgG subclass testing as well as the availability of corresponding data from female partners.

To conclude, we analyzed IgG1 and IgG3 antibody responses to 2 *C trachomatis* antigens, Pgp3 and Hsp60, as serological markers of past chlamydial infection and investigated their association with sperm quality among subfertile men. Our findings suggest that prior *C trachomatis* infection may adversely impact male fertility potential, likely due to its effects on sperm motility and morphology. Future studies, including prospective follow-up studies with larger study populations, will be needed to examine the role of *C trachomatis* and other sexually transmitted bacteria in adverse consequences of male reproductive health. Also, cell-mediated immunity should be studied in greater detail to elucidate the etiopathogenesis of *C trachomatis*–induced chronic inflammation in the male reproductive tract.
